# Mechanical ventilation and lung infection in the genesis of air-space enlargement

**DOI:** 10.1186/cc5680

**Published:** 2007-02-02

**Authors:** Alfonso Sartorius, Qin Lu, Silvia Vieira, Marc Tonnellier, Gilles Lenaour, Ivan Goldstein, Jean-Jacques Rouby

**Affiliations:** 1 Surgical Intensive Care Unit Pierre Viars, Department of Anesthesiology, Assistance Publique-Hôpitaux de Paris, La Pitié-Salpêtrière Hospital, 47-83 boulevard de l'Hôpital, 75013 Paris, France; 2 Department of Internal Medicine, Faculty of Medicine, Federal University from Rio Grande do Sul, Intensive Care Unit, Hospital de Clinicas de Porto Alegre, Rua Ramiro Barcelos, 2350 – 90035-903 Porto Alegre/Rio Grande do Sul, Brazil; 3 Medical Intensive Care Unit, Assistance Publique-Hôpitaux de Paris, La Pitié-Salpêtrière Hospital, 47-83 boulevard de l'Hôpital, 75013 Paris, France; 4 Department of Pathology, Assistance Publique-Hôpitaux de Paris, La Pitié-Salpêtrière Hospital, 47-83 boulevard de l'Hôpital, 75013 Paris, France

## Abstract

**Introduction:**

Air-space enlargement may result from mechanical ventilation and/or lung infection. The aim of this study was to assess how mechanical ventilation and lung infection influence the genesis of bronchiolar and alveolar distention.

**Methods:**

Four groups of piglets were studied: non-ventilated-non-inoculated (controls, *n *= 5), non-ventilated-inoculated (*n *= 6), ventilated-non-inoculated (*n *= 6), and ventilated-inoculated (*n *= 8) piglets. The respiratory tract of intubated piglets was inoculated with a highly concentrated solution of *Escherichia coli*. Mechanical ventilation was maintained during 60 hours with a tidal volume of 15 ml/kg and zero positive end-expiratory pressure. After sacrifice by exsanguination, lungs were fixed for histological and lung morphometry analyses.

**Results:**

Lung infection was present in all inoculated piglets and in five of the six ventilated-non-inoculated piglets. Mean alveolar and mean bronchiolar areas, measured using an analyzer computer system connected through a high-resolution color camera to an optical microscope, were significantly increased in non-ventilated-inoculated animals (+16% and +11%, respectively, compared to controls), in ventilated-non-inoculated animals (+49% and +49%, respectively, compared to controls), and in ventilated-inoculated animals (+95% and +118%, respectively, compared to controls). Mean alveolar and mean bronchiolar areas significantly correlated with the extension of lung infection (*R *= 0.50, *p *< 0.01 and *R *= 0.67, *p *< 0.001, respectively).

**Conclusion:**

Lung infection induces bronchiolar and alveolar distention. Mechanical ventilation induces secondary lung infection and is associated with further air-space enlargement. The combination of primary lung infection and mechanical ventilation markedly increases air-space enlargement, the degree of which depends on the severity and extension of lung infection.

## Introduction

Air-space enlargement is a prominent feature of ventilator-induced lung injury in patients with severe acute respiratory distress syndrome (ARDS). Emphysema-like lesions, bronchiectasis, and pseudocysts are frequently found at lung autopsy in patients ventilated over a long period of time [1-5]. Mechanical ventilation with high tidal volume and pressure is considered as a major cause of mechanical ventilation-induced lung injury [2,6]. Other mechanisms frequently encountered in the critical care environment, however, are likely to be involved in air-space enlargement: oxygen toxicity [7], prolonged exposure to nitric oxide [8], malnutrition [9], and chronic endotoxemia [10].

Ventilator-associated pneumonia is a common complication in patients receiving prolonged mechanical ventilation [11]. In an experimental model of severe bronchopneumonia, we demonstrated that significant air-space enlargement was observed after three days of mechanical ventilation using tidal volumes of 15 ml/kg and zero positive end-expiratory pressure (ZEEP) [12]. In that study, lung morphometry results were compared in mechanically ventilated piglets with and without inoculation pneumonia and it was therefore impossible to separate the effects of lung infection from those of mechanical ventilation in the genesis of bronchiolar and alveolar distention. In the present study, performed in the same experimental intensive care unit, lung morphometry was used for comparison between spontaneously breathing and mechanically ventilated piglets in order to assess how mechanical ventilation and lung infection influence air-space enlargement, respectively.

## Materials and methods

### Animal preparation

Twenty-five bred domestic Large White-Landrace piglets (three to four months old, weight 20 ± 2 kg) were anesthetized using propofol (3 mg/kg) and orotracheally intubated in the supine position. Anesthesia was maintained with a continuous infusion of midazolam (0.3 mg/kg per hour), pancuronium (0.3 mg/kg per hour), and fentanyl (5 μg/kg per hour). A catheter was inserted in the ear vein for continuous infusion of 10% dextrose and Ringer lactate, and the femoral artery was cannulated with a 3-French polyethylene catheter (Prodimed, Plastimed devision, Le Plessis-Bouchard, France) for pressure monitoring and blood sampling. All animals were treated according to the guidelines of the Department of Experimental Research of the Lille University (Lille, France) and to the *Guide for the Care and Use of Laboratory Animals *(National Institutes of Health publication no. 93-23, revised 1985).

### Mechanical ventilation management and bronchial inoculation

After technical preparation, the piglets were placed in the prone position that was maintained throughout the experiment. They were mechanically ventilated in a volume-controlled mode with a Cesar ventilator (Taema, Antony, France). The initial ventilator settings consisted of a tidal volume of 15 ml/kg, a respiratory rate of 15 breaths per minute, an inspiratory/expiratory ratio of 0.5, and ZEEP. Four groups of animals were studied: non-ventilated-non-inoculated (*n *= 5, controls), non-ventilated-inoculated (*n *= 6), ventilated-non-inoculated (*n *= 6), and ventilated-inoculated (*n *= 8) animals. Piglets of the control group were only anesthetized and ventilated for sacrifice. Non-ventilated-inoculated animals were ventilated for the bacterial inoculation and then extubated and kept in the animal house with free access to food until sacrifice 60 hours later. Ventilated-non-inoculated and ventilated-inoculated piglets were mechanically ventilated for a maximum of 60 hours or less if they died before. ZEEP was maintained and FiO_2 _(fraction of inspired oxygen) was increased in order to maintain arterial partial pressure of oxygen (PaO_2_) above 80 mm Hg, and PaCO_2 _(arterial partial pressure of carbon dioxide) was kept between 35 and 45 mm Hg by increasing the respiratory rate to the maximum level preceding the appearance of intrinsic positive end-expiratory pressure [13]. Above this limit, hypercapnia was tolerated. Peak and end-inspiratory plateau airway pressures were measured on the ventilator, and respiratory compliance was calculated by dividing the tidal volume by end-inspiratory pressure minus intrinsic positive end-expiratory pressure. Blood gases were analyzed at 37°C with an ABL120 blood gas analyzer (Radiometer A/S, Brønshøj, Denmark). Cardiorespiratory parameters were systematically recorded at six hour intervals.

By means of bronchoscopy, a suspension of *Escherichia coli *(10^6 ^colony-forming units [cfu] per milliliter, biotype 54465) was selectively inoculated in non-ventilated-inoculated and ventilated-inoculated piglets lying in the prone position. Forty milliliters was instilled in each lower lobe and 10 ml in each middle lobe.

### Fixation of the lungs

The piglets were sacrificed by exsanguination through direct cardiac puncture after sternotomy while maintaining mechanical ventilation. Following death, the left lung of ventilated-non-inoculated and ventilated-inoculated piglets and both lungs of control and non-ventilated-inoculated piglets were removed, weighed, and fixed at a lung volume close to the functional residual capacity (FRC). The lung was instilled step by step by a solution composed of formalin, ethanol, polyethylene glycol, and water. After each 50-ml instillation, the lung was replaced in the thorax to verify whether its volume fit the rib cage volume. If it did, instillation was stopped and the volume of instilled solution was considered as representative of FRC. The filling procedure was 30 cm H_2_O limited. After fixation, the lung was sagitally sectioned in the middle. The macroscopic aspect was carefully examined. Six blocks were sampled from upper, middle, and lower lobes for histological analysis [12]. Blocks were taken from dependent (ventral) and non-dependent (dorsal) sides of each lobe, and the distance between each block and the pulmonary apex was measured. The blocks were processed for routine histological preparation and embedded in paraffin. Sections of 4-μm thickness were cut and stained with hematoxylin and eosin.

### Collection of lung tissue specimens for bacteriological analysis

Following death, the right lungs of ventilated-non-inoculated and ventilated-inoculated piglets were removed and six lung tissue specimens (1 cm^3^) were excised from the non-dependent (dorsal) and dependent (ventral) segments of upper, middle, and lower lobes. Sampling was always performed in areas showing gross abnormalities when present. Quantitative bacterial analysis of lung bacterial burden was performed according to a previously described technique [14]. The total number of bacteria for each piglet was calculated by adding the absolute number of bacteria cultured from the specimen, and the result was expressed as colony-forming units per gram of tissue (cfu/g).

### Histological classification

Pneumonia was assessed on each secondary pulmonary lobule present in a given histological section and classified into five different categories as previously described [15,16]. Classification of a given pulmonary lobule was based on the worst category observed, and final classification for a segment was defined as the most frequently observed lesion in all secondary pulmonary lobules present in the histological sections cut from the tissue block. The percentage of each category was calculated as the number of secondary lobules of the category divided by the total number of lobules analyzed (multiplying the quotient by 100).

### Histomorphometry analysis of the lungs

Alveolar and bronchiolar areas were measured using a method previously described and an image-analyzer computerized system (Leica Q500IW, Leica Ltd, Cambridge, UK) coupled to a high-resolution color camera (JVC KYF 3 CCD; JVC, Yokohama, Japan) [12,17]. Alveolar dimensions were measured in lung areas remaining normally aerated. According to the extension of lung consolidation, 5 to 15 non-coincident aerated fields observed at a magnification of ×4 were analyzed on each histological section. Mean alveolar area was determined as the average area of the aerated alveoli present on all examined fields. Between 9 × 10^3 ^and 40 × 10^3 ^aerated alveoli were analyzed in each piglet. Bronchiolar dimensions were measured in all lung areas, either aerated or not. Mean bronchiolar area was defined as the mean area of the transversal section of non-cartilaginous bronchioles present on a given histological section. Between 220 and 270 bronchioles were analyzed in each piglet.

### Statistical analysis

Statistical analysis was performed using SigmaStat 2.03 software (SPSS Inc., Chicago, IL, USA). Data were expressed as mean ± standard deviation or as median and 25% to 75% interquartile range according to the data distribution. Cardiorespiratory parameters between four groups were compared by an analysis of variance followed by a protected least significance Fisher exact test. Regional distributions of mean alveolar and bronchiolar areas of each group were compared by a two-way analysis of variance for two factors (lobes and dependence of the lung) followed by a post hoc analysis (Holm-Sidak test). The presence of a significant interaction indicates that the regional distribution of mean alveolar or bronchiolar areas in upper, middle, and lower lobes was different between non-dependent (dorsal) and dependent (ventral) lung regions. The differences of mean alveolar areas and mean bronchiolar areas between the groups were compared by a non-parametric Kruskal-Wallis test followed by a post hoc Dunn's analysis. The percentage of infected secondary lobules in the different groups was compared by χ^2 ^test. Correlations were made by linear regression analysis. Statistical significance level was fixed at 0.05.

## Results

### Animals

Clinical characteristics of the four groups of piglets are summarized in Table 1. Five ventilated-inoculated piglets died before the end of the protocol, two from compressive pneumothorax and three from septic shock confirmed by positive blood cultures, thereby reducing the preset duration of mechanical ventilation. As shown in Table 2, PaO_2 _and mean arterial pressure before death were significantly lower in ventilated-non-inoculated and ventilated-inoculated animals than in control piglets. Respiratory compliance was significantly lower in ventilated-inoculated animals than in control piglets. In addition, the exsanguinated lung weight was significantly higher and FRC was lower in ventilated-inoculated piglets than in control animals (Table 1).

**Table 1:** Clinical characteristics of the four groups of piglets

Clinical characteristic	Group				*p *value
	Control	NVI	VNI	VI	

Number of animals	5	6	6	8	
Weight (kg)	21 ± 2	19 ± 1	22 ± 2	22 ± 2	NS
Duration of mechanical ventilation (minutes)			3,600	2,505 ± 883	
Ventilation for inoculation (minutes)		144 ± 43			
Ventilation for sacrifice (minutes)	28 ± 3	8 ± 6			
Inoculum of *Escherichia coli *(cfu/ml)	0	10^6^	0	10^6^	
Infected piglets (*n*)	0	6	5	8	
Death before the end of the protocol (*n*)	0	0	0	5	
Functional residual capacity (ml)	331 ± 51	303 ± 52	240 ± 90	184 ± 97^a,b^	<0.05
Weight of lung after death (g)	97 ± 22^b^	128 ± 10	160 ± 64^a^	281 ± 105^a,b^	<0.01

^a^*p *< 0.05 versus control; ^b^*p *< 0.05 versus NVI. Data are expressed as mean ± standard deviation. cfu, colony-forming units; NS, not significant; NVI, non-ventilated-inoculated piglets; VI, ventilated-inoculated piglets; VNI, ventilated-non-inoculated piglets.

**Table 2:** Cardiorespiratory parameters measured in the four groups of piglets before death

Cardiorespiratory parameters	Group				*p *value
	Control	NVI	VNI	VI	
Number of animals	5	6	6	8	

pH	7.41 ± 0.04	7.38 ± 0.04	7.40 ± 0.27	7.40 ± 0.10	0.12
PaO_2_/FiO_2 _(mm Hg)	414 ± 82	385 ± 38	279 ± 130^a^	208 ± 113^b,c^	<0.001
PaCO_2 _(mm Hg)	37 ± 6	41 ± 3	40 ± 7	44 ± 7	0.16
Ppeak (cm H_2_O)	19 ± 1	NP	28 ± 14	37 ± 14	0.05
Pplat (cm H_2_O)	15 ± 3	NP	20 ± 11	27 ± 10	0.07
Crs (ml/cm H_2_O)	25 ± 7	NP	20 ± 6	16 ± 6^a^	<0.05
MAP (mmHg)	116 ± 11	106 ± 11	92 ± 12^a^	74 ± 24^b,c^	0.001

^a^*p *< 0.05 versus control; ^b^*p *< 0.01 versus control; ^c^*p *< 0.05 versus NVI. Data are expressed as mean ± standard deviation. Crs, respiratory compliance; MAP, mean arterial pressure; NP, not performed; NVI, non-ventilated-inoculated piglets; PaCO_2_, arterial partial pressure of carbon dioxide; PaO_2_/FiO_2_, arterial partial pressure of oxygen/fraction of inspired oxygen; Ppeak, maximum peak airway pressure; Pplat, end-inspiratory plateau airway pressure; VI, ventilated-inoculated piglets; VNI, ventilated-non-inoculated piglets.

### Histological and bacteriological characteristics of lung infection

**Figure 1:**
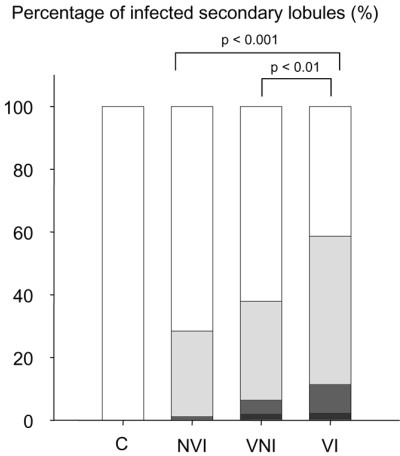
Severity of lung infection in the four groups of piglets. Data are expressed as the percentage of infected secondary pulmonary lobules corresponding to a given category of pneumonia. White bar represents healthy lung, light gray bar represents focal pneumonia, dark gray bar represents confluent pneumonia, and black bar represents purulent pneumonia. C, control piglets; NVI, non-ventilated-inoculated piglets; VI, ventilated-inoculated piglets; VNI, ventilated-non-inoculated piglets.

Severity of lung infection is shown in Figure 1. All secondary pulmonary lobules of control animals were free of pathological findings. In non-ventilated-inoculated piglets, 28% of secondary pulmonary lobules were infected: 27% with focal and 1% with confluent pneumonia. In ventilated-non-inoculated piglets, 38% of secondary pulmonary lobules were infected: 31% with focal, 5% with confluent, and 2% with purulent pneumonia, the lung infection predominating in dependent (ventral) compared to non-dependent (dorsal) lung regions (*p *< 0.05). A single piglet was free of any histological lung infection. In ventilated-inoculated piglets, 58% of secondary lobules were infected: 47% with focal, 9% with confluent, and 2% with purulent pneumonia. As expected, lung infection was more extensive in ventilated-inoculated piglets than in ventilated-non-inoculated piglets (Figure 1). Similarly, lung infection was more extensive in ventilated-inoculated piglets than in non-ventilated-inoculated piglets. Isolated bronchiolitis represented less than 1% of pulmonary lobules in each of the four groups.

Bacteria predominantly found in the lung tissue specimens of ventilated-non-inoculated animals and ventilated-inoculated animals and their respective ranges were *E. coli *(0 to 10^4 ^versus 10^4 ^to 2 × 10^8 ^cfu/g), *Pasteurella multocida *(0 to 2 × 10^4 ^versus 0 to 2 × 10^6 ^cfu/g), *Pseudomonas aeruginosa *(0 to 2 × 10^3 ^versus 0 to 6 × 10^4 ^cfu/g), and *Streptococcus suis *(0 to 5 × 10^4 ^versus 6 to 6 × 10^6 ^cfu/g). Significantly higher bacterial concentrations were observed in the ventilated-inoculated group.

### Effects of mechanical ventilation and lung infection on air-space enlargement

**Figure 2:**
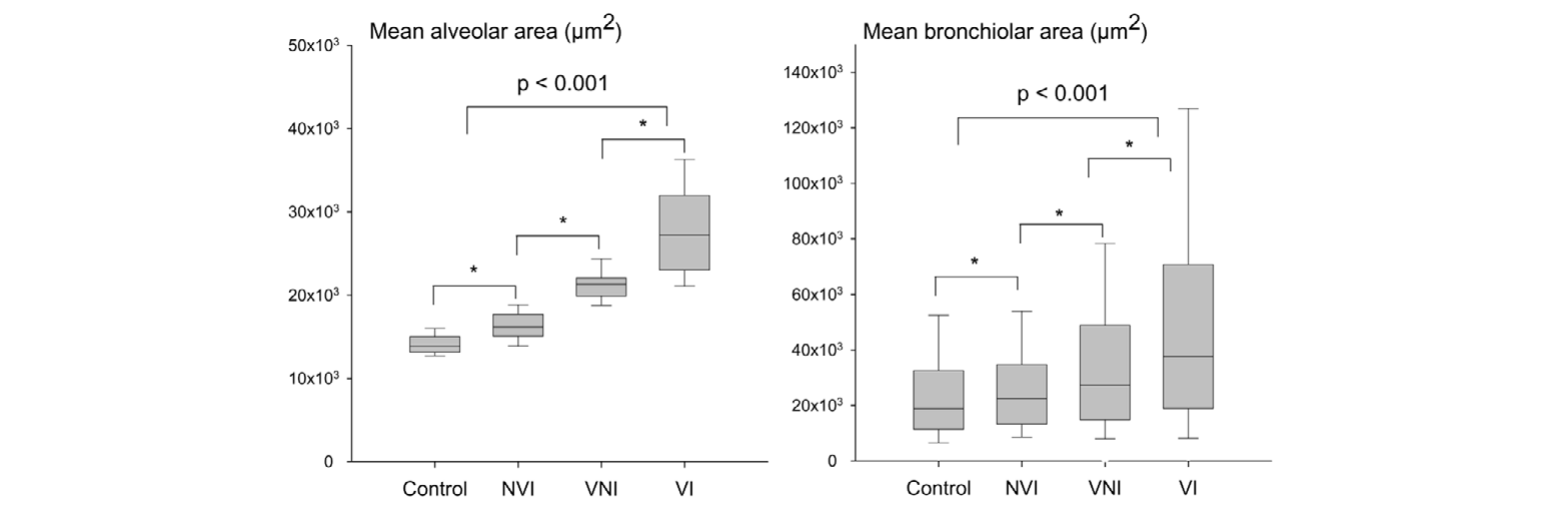
Mean alveolar and mean bronchiolar areas in the four groups of piglets. Mean alveolar area was measured in aerated lung regions (left panel), and mean bronchiolar area was measured in aerated and non-aerated lung regions (right panel). Data are expressed as median and 25% to 75% interquartile range. *p < 0.05 between two groups. NVI, non-ventilated-inoculated piglets; VI, ventilated-inoculated piglets; VNI, ventilated-non-inoculated piglets.

**Figure 3:**
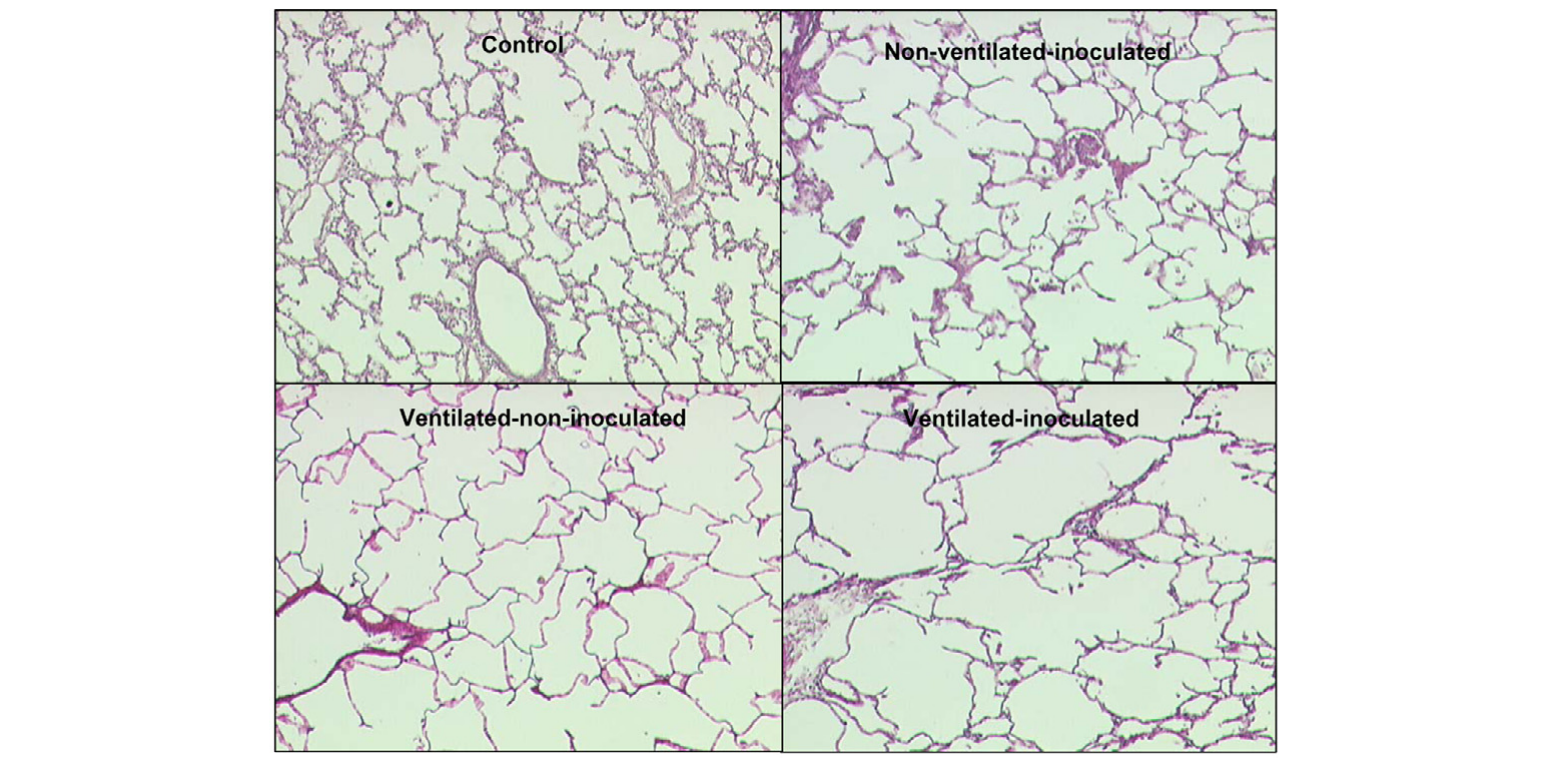
Histological evidence of alveolar overinflation caused by mechanical ventilation and lung infection. The different histological sections (magnification ×4) are representative of aerated lung regions of a control piglet, a non-ventilated-inoculated piglet, a ventilated-non-inoculated piglet, and a ventilated-inoculated piglet. Mean alveolar area is increased in the three study groups. In ventilated-non-inoculated animals, a stretching of alveolar walls is observed. Alveolar distortion is amplified when lung infection and mechanical ventilation are associated.

Mean alveolar area was significantly greater in the three experimental groups of piglets than in control animals (Figure 2): +16% in non-ventilated-inoculated animals, +49% in ventilated-non-inoculated animals, and +95% in ventilated-inoculated animals. Differences between the groups were significant (*p *< 0.001). In the single ventilated-non-inoculated piglet without lung infection, mean alveolar area was 21,639 ± 27,730 μm^2^. As a comparison, the mean alveolar area observed in the control group was 14,145 ± 14,271 μm^2^. Figure 3 shows histological sections illustrative of alveolar distention present in aerated-non-infected lung regions of an individual animal representative of each group.

**Figure 4:**
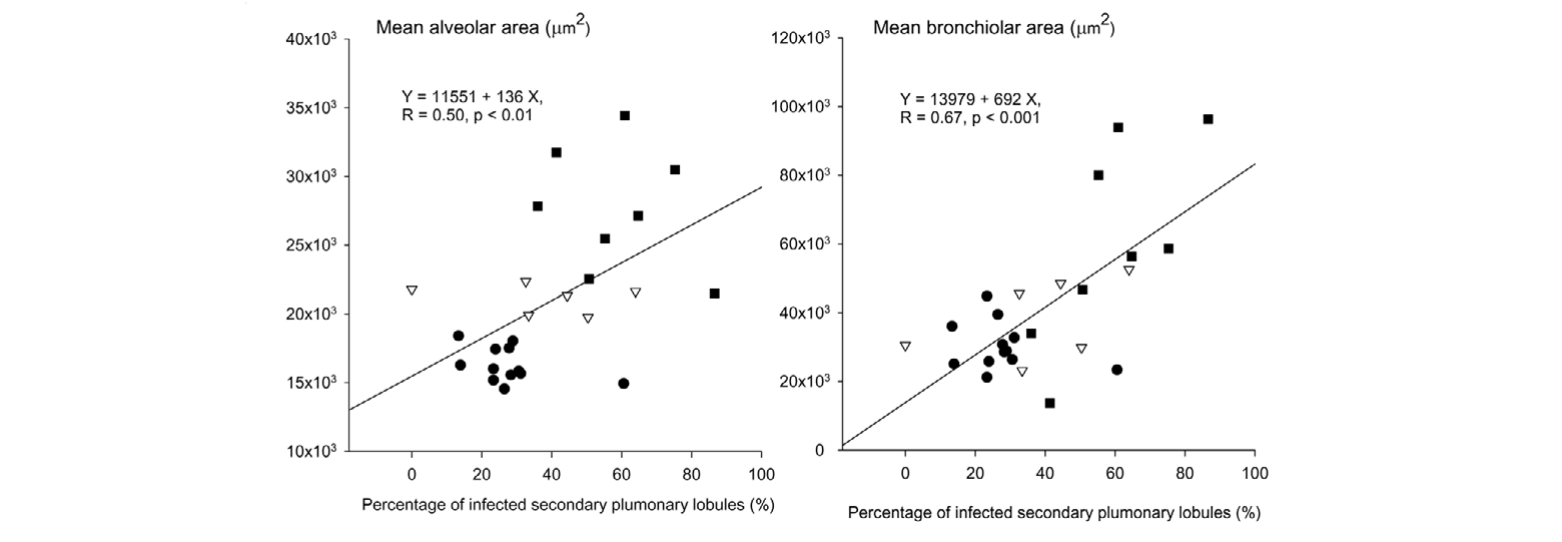
Correlations between percentages of infected secondary pulmonary lobules, mean alveolar area, and mean bronchiolar area. Closed circles, open triangles, and closed squares represent non-ventilated-inoculated piglets, ventilated-non-inoculated piglets, and ventilated-inoculated piglets, respectively. Each symbol is representative of one lung per animal except for non-ventilated-inoculated piglets, which are represented by two circles, each one corresponding to the right or left lung.

Mean bronchiolar area was significantly greater in non-ventilated-inoculated, ventilated-non-inoculated, and ventilated-inoculated animals than in control piglets (+11%, +49%, and +118%, respectively). Differences between the groups were statistically significant (*p *< 0.001). In the single ventilated-non-inoculated piglet free of any lung infection, bronchiolar area was 29,041 ± 22,156 μm^2^. As a comparison, the mean bronchiolar area observed in the control group was 25,395 ± 21,645 μm^2^. Mean alveolar and bronchiolar areas correlated linearly with the percentage of infected secondary pulmonary lobules (Figure 4).

### Regional distribution of bronchiolar and alveolar distention

**Figure 5:**
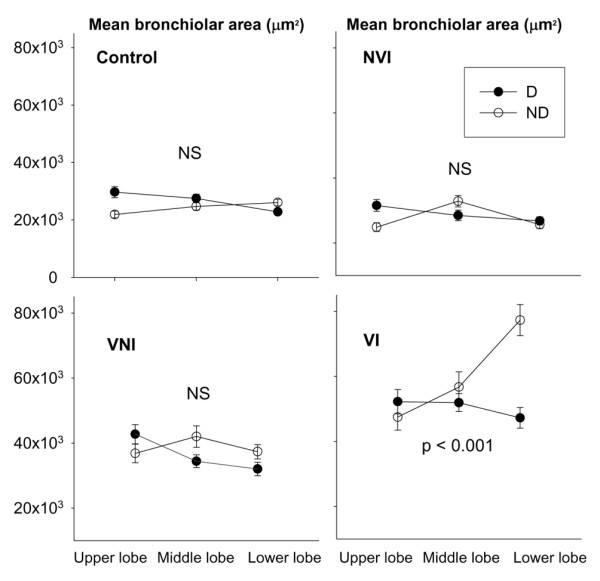
Regional distribution of mean bronchiolar area in the four groups of piglets. Data were expressed as mean ± standard error of the mean. *P *< 0.001 = interaction between lobes and dependence of the lung. D, dependent regions; ND, non-dependent regions; NS, not significant; NVI, non-ventilated-inoculated piglets; VI, ventilated-inoculated piglets; VNI, ventilated-non-inoculated piglets.

The increase in mean alveolar area was homogeneously distributed in the three groups of animals. As shown in Figure 5, the increase in mean bronchiolar area predominantly involved massively infected, non-dependent (dorsal) regions of lower lobes of ventilated-inoculated piglets.

## Discussion

The major findings of this study are (a) in spontaneously breathing animals, inoculation pneumonia induces moderate air-space enlargement and (b) in animals on prolonged and injurious mechanical ventilation, inoculation pneumonia induces severe air-space enlargement resulting in distortion of lung parenchyma structures. Because all but one ventilated-non-inoculated animal had evidence of ventilator-associated pneumonia at the end of the experiment (although the single animal presented obvious air-space enlargement), the present study does not allow us to conclude whether mechanical ventilation without associated lung infection produces bronchiolar and alveolar enlargement.

### Lung infection and mechanical ventilation-induced air-space enlargement

An original finding of the study is that, in spontaneously breathing inoculated piglets, alveolar spaces of lung regions unaffected by the infectious process were significantly enlarged compared to alveoli of control animals. One of the possible explanations is that lung infection damages the matrix of alveolar walls by promoting the release of metalloproteinases by activated neutrophils [18,19]. Lung matrix metalloproteinases are degradative enzymes that reduce the densities of collagen, fibronectin, and elastin [20]. In patients with hospital-acquired pneumonia, high concentrations of matrix metalloproteinases are found in bronchoalveolar lavage, the level of which is highly correlated with the severity of lung infection [21]. It has also been suggested that metalloproteinases could be involved in the genesis of bronchiectasis [22].

A substantial bronchiolar dilatation was evidenced in ventilated animals with either ventilator-associated pneumonia or severe inoculation pneumonia: the bronchiolar dilatation was found preferentially in massively infected, non-dependent (dorsal) parts of lower lobes. Piglets are four-legged animals and their physiological position is the prone position, during which ventral segments are 'dependent.' In patients lying in the supine position, ventral segments of lower lobes are non-dependent. Therefore, the regional distribution found in piglets is very similar to the distribution of lung overinflation reported in patients in the early stage of ARDS: in the supine position, lung overinflation is found in caudal and non-dependent lung regions (middle lobe and ventral segment of lower lobes) [23]. At late stages, pseudocysts and bronchiectasis found on computed tomography demonstrate a nearly identical distribution [24]. From histological findings of the present study, it can be reasonably hypothesized that lung overinflation and air cysts found in the early and late stages of ARDS are, at least partially, of bronchial origin.

The regional distribution of bronchiolar dilatation pleads for a direct mechanical stress resulting from positive pressure. When lung infection is found predominantly in dependent parts of the lung, tidal volume is preferentially distributed in non-dependent lung regions with persisting lung aeration. In ventilated-inoculated piglets, lung infection involved more than two thirds of the lungs and the tidal volume of 15 ml/kg delivered to the lung spared by the infectious process was equivalent to a tidal volume of 45 ml/kg delivered to a healthy lung.

### Interactions between mechanical ventilation, lung infection, and air-space enlargement

The reported incidence of ventilator-associated pneumonia in mechanically ventilated patients ranges between 20% and 60% and is highly dependent upon diagnostic tools [25,26]. Experimentally, more than 90% of anesthetized baboons and piglets ventilated beyond 2 days show histological evidence of ventilator-associated pneumonia [27-30]. In confirmation of these previous experiments, five of six piglets with initially healthy lung had bacteriological and histological evidence of lung infection after 60 hours of mechanical ventilation. As previously demonstrated [15,16,30], ventilator-associated pneumonia predominated in dependent lung regions. Although data are lacking in humans, it is highly likely that similar events occur in patients on prolonged mechanical ventilation. Therefore, mechanical ventilation-induced air-space enlargement results in the majority of cases from the interaction between infection and mechanical ventilation and our model is clinically relevant. Such a high incidence of ventilator-associated pneumonia prevents us from determining with certainty whether mechanical ventilation with a tidal volume of 15 ml/kg by itself produces alveolar enlargement independently of lung infection. The deleterious role of mechanical ventilation on air-space enlargement, however, is clearly shown in the single piglet without any detectable lung infection but presenting histological evidence of alveolar enlargement. The light-microscopic analysis showed diffusely distributed alveolar and bronchiolar enlargement with stretched alveolar walls and presence of moderate interstitial and peribronchiolar edema. These latter histological findings were observed previously at the early phase of ventilator-induced lung injury in both small and large animals after a short period of mechanical ventilation delivering high-peak airway pressure or high tidal volume [31,32].

In confirmation of a recent experimental study [33], pneumonia was more extensive and severe in mechanically ventilated than in spontaneously breathing animals for the same bacterial inoculation. Furthermore, our bacteriological results demonstrated that, in ventilated-inoculated animals, *E. coli *(the inoculated bacteria) was frequently associated with *P. aeruginosa*, *S. suis*, and *P. multocida*, all of which attest the presence of ventilator-associated pneumonia. Therefore, in this group of animals, lung infection was more severe and extensive than in ventilated-non-inoculated animals. As a consequence, alveolar and bronchiolar dilatations that correlate positively with the extension of lung infection were amplified after 60 hours of mechanical ventilation.

Finally, lung infection and mechanical ventilation may act synergistically in the genesis of air-space enlargement. A genuine vicious circle is produced. As recently demonstrated by Whitehead and colleagues [34], a high tidal volume ventilation can markedly reduce the release of inflammatory cytokines in response to intratracheal lipopolysaccharide. This paradoxical result seems to be related to a reduction of the alveolar macrophage population, an effect that could increase susceptibility to infection and amplify ventilator-induced lung injury [35].

Lung infection damages the extracellular matrix of alveolar walls by increasing lung proteolytic activity and reduces lung aeration. The resulting increased mechanical stress exerted on non-infected lung areas amplifies the lung distention resulting from collagen and elastin degradation. It also produces a mechanical dilatation of bronchioles within pneumonic areas [12]. In addition, it has been shown that the combination of mechanical ventilation and acute endotoxemia may distend previously healthy lung areas [36]. Air-space enlargement may also result from prolonged exposure to high oxygen concentrations [7], malnutrition [9], and chronic endotoxemia [10], all factors frequently observed in critically ill patients. With time, lung overinflation and distortion involve significant parts of the lung [2,16], increase alveolar dead space [37], and become apparent on lung computed tomography [23,24].

### Methodological limitations

The main objective of the study was to evaluate the respective roles of lung infection and mechanical ventilation in the genesis of air-space enlargement. Therefore, a model consisting of ventilating animals with a tidal volume of 15 ml/kg and ZEEP was selected in order to reproduce air-space enlargement previously observed [12]. Our findings may not apply to patients ventilated with low tidal volume and positive end-expiratory pressure [35,38]. Recent experimental studies have indirectly suggested that reducing tidal volume reduces lung injury resulting from lung infection [39,40]. Further experimental studies are required to assess whether the combination of optimizing ventilatory strategy by reducing tidal volume and applying positive end-expiratory pressure attenuates air-space enlargement resulting from mechanical ventilation and lung infection.

The technique used for lung fixation is another factor that could have influenced the morphometry results. Although the lung was slowly instilled in 50-ml increments to reach a pulmonary volume close to the actual disease-related FRC, the artifactual overinflation of non-infected lung areas cannot be totally ruled out. In ventilated-inoculated piglets, 60% of the lung was massively infected and non-aerated, whereas 40% remained normally or partially aerated. The latter regions were exposed to the risk of overinflation during the filling procedure, exactly as during tidal inflation in patients on mechanical ventilation. The filling procedure, however, was performed step by step outside the thorax and each 50-ml inflation was followed by the repositioning of the lung within the rib cage until a perfect fitting was obtained. These two methodological elements very likely reduced the risk of artifactual overinflation considerably.

For mimicking of clinical conditions, ventilated-non-inoculated and spontaneously breathing animals rather than ventilated-saline-inoculated and spontaneously breathing animals were chosen as controls in the present study. It could be hypothesized that liquid instillation by itself may induce mechanical airway plugging that results in dependent atelectasis and overinflation of non-dependent units. Based on previous experimental studies demonstrating that bronchoalveolar lavage less than or equal to 4 ml/kg [41,42] does not induce significant histological injury, the volume of bacterial suspension instilled into the lung was substantially reduced in order to minimize a plugging effect.

## Conclusion

Experimental lung infection produces bronchiolar and alveolar enlargement. If lung infection is combined with mechanical ventilation, air-space enlargement is markedly amplified. The degree of air-space enlargement depends on the severity and extension of lung infection. It is, however, virtually impossible to distinguish the respective roles of lung infection and mechanical ventilation in the genesis of air-space enlargement because secondary lung infection occurs nearly constantly after a few days of mechanical ventilation. Additional experimental studies are required to assess whether the combination of reducing tidal volume to 6 ml/kg and applying different levels of positive end-expiratory pressure attenuates air-space enlargement resulting from mechanical ventilation and lung infection.

## Key messages

• Lung infection induces bronchiolar and alveolar distention.

• Mechanical ventilation rapidly induces secondary lung infection and air-space enlargement.

• The association of lung infection and mechanical ventilation markedly increases air-space enlargement.

## Abbreviations

ARDS = acute respiratory distress syndrome; cfu = colony-forming units; FRC = functional residual capacity; PaO_2 _= arterial partial pressure of oxygen; ZEEP = zero positive end-expiratory pressure.

## Competing interests

The authors declare that they have no competing interests.

## Authors' contributions

AS and QL carried out the study and drafted the manuscript. SV, MT, and IG participated in the study and study analysis. GL participated in the histological section preparation and histomorphometry analysis. J-JR participated in the design of the study and helped to draft the manuscript. All authors read and approved the final manuscript.
